# Value of quantitative analysis in lung computed tomography in patients severely ill with COVID-19

**DOI:** 10.1371/journal.pone.0251946

**Published:** 2021-05-20

**Authors:** Marta Rorat, Tomasz Jurek, Krzysztof Simon, Maciej Guziński

**Affiliations:** 1 Department of Forensic Medicine, Wroclaw Medical University, Wroclaw, Poland; 2 Department of Infectious Diseases and Hepatology, Wroclaw Medical University, Wroclaw, Poland; 3 Department of General Radiology, Interventional Radiology and Neuroradiology, Wroclaw Medical University, Wroclaw, Poland; Kaohsuing Medical University Hospital, TAIWAN

## Abstract

**Introduction:**

Quantitative computed tomography (QCT) is used to objectively assess the degree of parenchymal impairment in COVID-19 pneumonia.

**Materials and methods:**

Retrospective study on 61 COVID-19 patients (severe and non-severe; 33 men, age 63+/-15 years) who underwent a CT scan due to tachypnea, dyspnoea or desaturation. QCT was performed using VCAR software. Patients’ clinical data was collected, including laboratory results and oxygenation support. The optimal cut-off point for CT parameters for predicting death and respiratory support was performed by maximizing the Youden Index in a receiver operating characteristic (ROC) curve analysis.

**Results:**

The analysis revealed significantly greater progression of changes: ground-glass opacities (GGO) (31,42% v 13,89%, p<0.001), consolidation (11,85% v 3,32%, p<0.001) in patients with severe disease compared to non-severe disease. Five lobes were involved in all patients with severe disease. In non-severe patients, a positive correlation was found between severity of GGO, consolidation and emphysema and sex, tachypnea, chest x-ray (CXR) score on admission and laboratory parameters: CRP, D-dimer, ALT, lymphocyte count and lymphocyte/neutrophil ratio. In the group of severe patients, a correlation was found between sex, creatinine level and death. ROC analysis on death prediction was used to establish the cut-off point for GGO at 24.3% (AUC 0.8878, 95% CI 0.7889–0.9866; sensitivity 91.7%, specificity 75.5%), 5.6% for consolidation (AUC 0.7466, 95% CI 0.6009–0.8923; sensitivity 83.3%, specificity 59.2%), and 37.8% for total (GGO+consolidation) (AUC 0.8622, 95% CI 0.7525–0.972; sensitivity 75%, specificity 83.7%). The cut-off point for predicting respiratory support was established for GGO at 18.7% (AUC 0.7611, 95% CI 0.6268–0.8954; sensitivity 87.5%, specificity 64.4%), consolidation at 3.88% (AUC 0.7438, 95% CI 0.6146–0.8729; sensitivity 100%, specificity 46.7%), and total at 23.5% (AUC 0.7931, 95% CI 0.673–0.9131; sensitivity 93.8%, specificity 57.8%).

**Conclusion:**

QCT is a good diagnostic tool which facilitates decision-making regarding intensification of oxygen support and transfer to an intensive care unit in patients severely ill with COVID-19 pneumonia. QCT can make an independent and simple screening tool to assess the risk of death, regardless of clinical symptoms. Usefulness of QCT to predict the risk of death is higher than to assess the indications for respiratory support.

## Introduction

The appearance and severity of pneumonia are the main determinants for the diagnosis and further management of patients with severe acute respiratory syndrome coronavirus 2 (SARS-CoV-2). In literature a lot of attention has been paid to predictor values of various lab parameters in patients with COVID-19 [1–3]. One of the key diagnostic tools in COVID-19 pneumonia and its complications is computed tomography (CT) imaging [4–6]. CT is the most sensitive radiological technique for COVID-19 diagnosis with its sensitivity exceeding 90%, and false-negative results mostly involving patients who are symptomatic for less than 3 days [7], The most typical CT features of COVID-19 pneumonia are bilateral, peripheral, subpleural and multifocal (usually in five lobes) ground-glass opacities (GGO). Other changes include thin reticulations, peribronchovascular thickening, vascular dilatations within pneumonia areas or architectural distortion. The following progressions are observed within the course of the disease: lung lesions in extent and attenuation value and evolution towards crazy-paving pattern areas or linear and retractile consolidation areas. Maximum lung damage is usually observed around day 10 and then gradually decreases [7].

The benefit of CT is not only a precise assessment of lesions and their distribution but also the possibility of quantitative assessment of the volume of lung parenchyma involved. After traditional semi-quantitative methods based on visual scoring, more and more attention is being paid to quantitative analysis of CT images, which present better performance and efficiency [8]. The total extent of lung involvement as well as the density of pulmonary lesions on the CT examination are markers of disease severity [9, 10]. The French Society of Thoracic Imaging’s recommendations grading lung involvement, based on visual assessment, are as follows: absent or minimal (< 10%), moderate (10–25%), extensive (25–50%), severe (50–75%) or critical (> 75%) [11].

15 to 30% of hospitalised patients progress to acute respiratory distress syndrome (ARDS), which is characterised on the CT by extensive bilateral lung consolidation and is associated with poor prognosis [7, 11]. Invasive mechanical ventilation of patients admitted to the intensive care unit (ICU) ranges from 29.1 to 89.9% and possible mortality from 24.5 to 96.7% in different countries [12]. Computer analysis of CT image using the program for quantitative assessment of parenchymal involvement can be used to evaluate the severity of the disease which is important in assessing the prognosis of COVID-19 and the need for respiratory ventilation [9, 11]. Being able to assess the degree of parenchymal involvement, we are able to take decisions to intensify therapies or even to predict the risk of death.

The aim of this study is to determine the value of compromised lung volume analysis in making decisions about intensifying the treatment of respiratory failure and predicting the risk of death in the course of COVID-19 in correlation with selected clinical parameters.

## Materials and methods

This is a retrospective analysis of the medical records of 448 consecutive patients hospitalised due to COVID-19 in Gromkowski Specialist Regional Hospital in Wroclaw (Poland) between 25 March and 6 August 2020. All data was accessed between 20th August and 12th September, fully anonymized before access, and the local ethics committee waived the requirement to obtain informed consent to process data from patients’ medical records used in the research. All cases were confirmed by reverse transcription–polymerase chain reaction (RT-PCR) test. We chose 70 patients (severe and non-severe; 33 men, age 63+/-15 years) in which:

1. Clinical features of pneumonia were found
2. Chest X-ray (CXR) confirmed pneumonia
3. A chest CT was performed during deterioration of health (presence of dyspnoea, respiratory effort, symptoms of respiratory failure or its exacerbation, disorder of consciousness, sudden increase of the parameters of inflammation, an increase of D-dimers level.)

**Fig 1:**
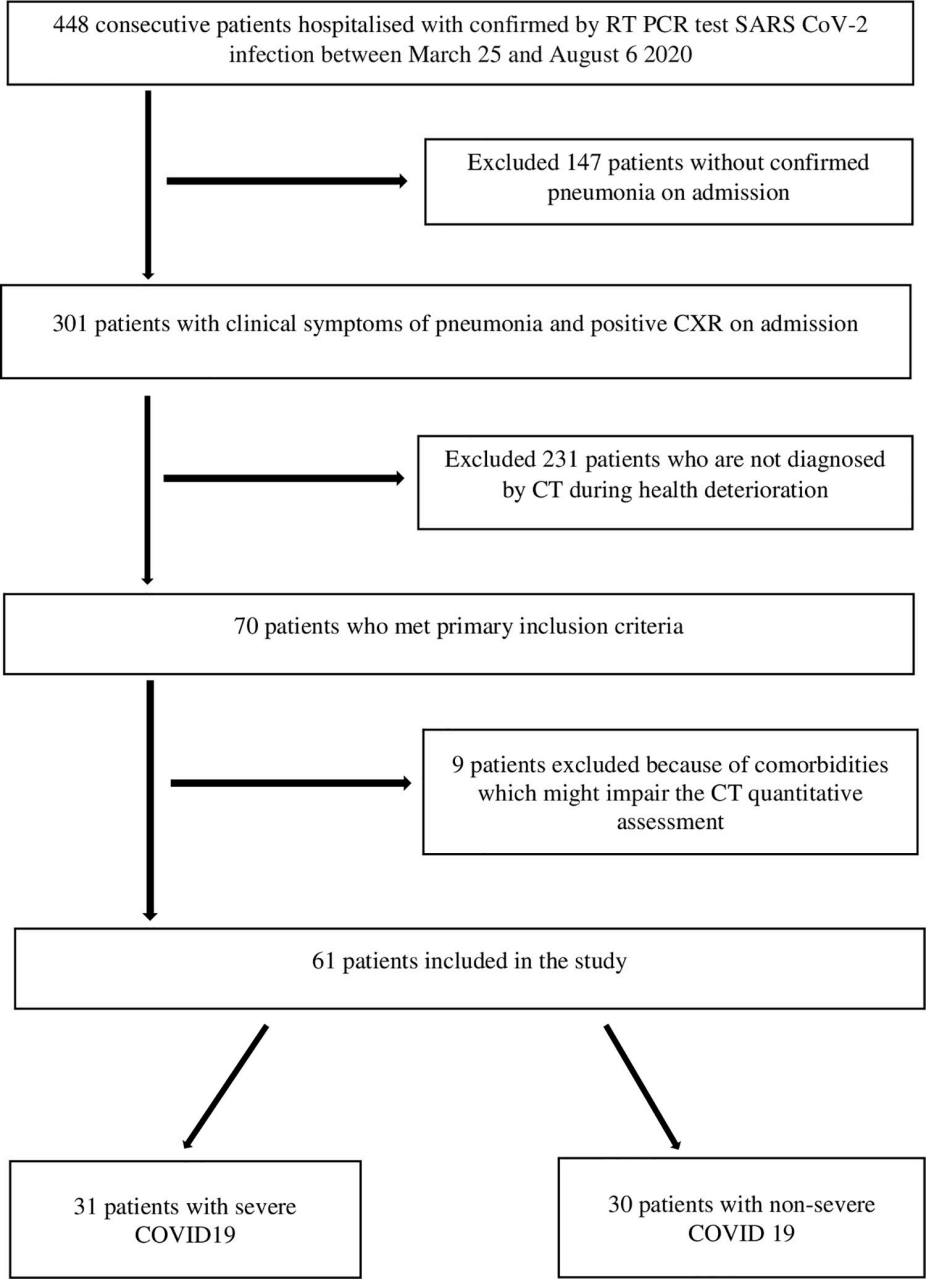
Flowchart of inclusion on the patients.

9 out of 70 patients, who had active tuberculosis, lung cancer, lung fibrosis and advanced chronic obstructive pulmonary disease, the patients without features of pneumonia on CT scan (ground-glass opacities +/- consolidation) or with poor quality of CT scan were excluded from the research. Finally 61 patients were included for further analysis. (Fig 1)

The patients were divided into two groups: severe (= severe pneumonia) and non-severe (non-severe pneumonia). Severe pneumonia was defined according to the WHO definition: person with clinical signs of pneumonia (fever, cough, dyspnoea, rapid breathing) plus one of the following: respiratory rate >30 breaths/min; severe respiratory distress; or SpO_2_ <90% with room air [13].

Chest CT examinations during inspiration were performed using a 64-slice CT scanner in tube potential, 120 kVp; detector configuration, 64 x 0.625mm; rotation time 0.4 s. [PHILIPS INGENUITY CORE 64]. All patients getting a CT scan as a standard basis for COVID-19 and the clinical benefits outweigh the risks of high ionization exposure. Quantitative modelling of thin-section chest CT without intravenous contrast agents by board-certificated radiologist with 9 years of experience in interpreting lung CT was performed. CT features of pneumonia lesions were automatically calculated using Thoracic VCAR software [GE Healthcare, USA], representing the percentages of ground-glass opacity volume, consolidation volume and emphysema volume in both lungs. Total lesion calculation was also performed, which made a total of ground-glass opacity and consolidation volumes. Thoracic VCAR provided automatic segmentation of the lungs. Segmentation of the lungs was performed using adaptive density-based morphology. The lungs were extracted by using an optimal thresholding to identify low-density fields in the scans, region growing (automating seed generation method to segment an image into regions with respect to a set of seeds) and void filling. The three-dimensional hole filling was used to fill the lung cavities created by the elimination of normal blood vessels during the thresholding process while airways are automatically segmented and exempted by iterative application of increasingly restrictive constraints to a thresholding and 3D region growing process. The software complies with the regulatory requirements of Council Directive 93/42/EEC concerning medical devices (CE 0459) and FDA regulations. Lung parenchyma was divided by Hounsfield unit (HU) intervals from -1024 HU to less than -950 HU representing emphysematous changes, values from higher than -950 HU to -700 HU representing normal parenchyma, values from -700 HU to -300 HU representing ground glass opacity (GGO), values from higher than -300 HU to 0 HU representing reticular opacity and some other denser fibrotic changes including honeycombing and nodules. In exact tracking or segmentation can be translated into imprecise quantitative measures. Twelve examinations (19.7%) required manual adjustment after automatic segmentation of the lungs by Thoracic VCAR by adding (miss apex or basal segment in nine cases) or removing extra structures (soft tissue: liver or mussels in three cases). Three examinations with inaccurate segmentation due to shallow inspiration acquisition and eccentric lung positioning were excluded from the study.

The study report, in addition to the CT results, comprised: information on the patients’ medical history, symptoms and their duration (measured from the time of onset of the first symptoms until performing the first CT scan), oxygen saturation, changes in chest radiography (CXR) assessed with five-point severity scoring system [1, 14], laboratory tests: morphology, C-reactive protein, procalcitonin, creatinine, lactate dehydrogenase (LDH), alanine aminotransferase (ALT), D–dimer and ferritin levels. For the analysis those laboratory parameters were selected which–according to the literature, have prognostic value for COVID-19. All laboratory tests were performed on the same day as the CT imaging.

### Statistics

Comparison of the severe and non-severe group using CT, demographics, clinical condition and laboratory results was performed using the Mann-Whitney test, chi-square test or Fisher’s exact test. The association between CT parameters and other variables (duration of symptoms, demographic, clinical condition and laboratory results) was assessed using the Mann-Whitney (in comparison of two groups) or Spearman’s rank correlation coefficient (in assessment of correlation between two quantitative variables). The optimal cut-off point for CT parameters for predicting death in both severe and not-severe groups was performed by maximising the Youden Index in a receiver operating characteristic curve analysis. For all statistical tests, the P-value <0.05 was considered significant. All tests were two tailed. Calculations were performed using the R statistical program for Windows (version 4.0.2) [15].

## Results

The baseline characteristics of the studied patients are described in Table 1. This data was used to demonstrate features between severe and non-severe groups. In the group of patients with severe disease, the mean age was 9 years higher than in the group with non-severe disease; hypertension and diabetes were more prevalent, tachypnea was observed more often, the changes in chest radiography on admission were more intense, oxygen saturation was decreased, the patients had higher values of CRP, PCT, D-dimers, LDH, lower lymphocyte counts and lymphocyte/neutrophil index. In this group, patients required a transfer to the intensive care unit and respiratory support (mainly mechanical ventilation) more often and had higher mortality. The results of the above mentioned analysis indicate that the criteria used to qualify patients for the research were correct. Table 2 shows the results of the quantitative analysis of CT images.

**Table 1:** The baseline characteristics of the studied patients.

Characteristic	Non-severe	Severe	P-value
	N = 30 (49%)	N = 31 (51%)	
Gender			
Male	15 (50%)	18 (58%)	0,700
Female	15 (50%)	13 (42%)	
Age (y), mean (SD); range	58 (12); 50–68	67 (17); 59–80	0,020*
Concomitant diseases			
Hypertension	10 (33%)	20 (65%)	0.029*
Cardiovascular diseases	7 (23%)	13 (42%)	0,200
Pulmonary diseases	2 (6,7%)	2 (6,5%)	*>*0,900
Malignant neoplasm	2 (6,7%)	4 (13%)	0,700
Obesity	5 (17%)	10 (32%)	0,300
Diabetes	3 (10%)	12 (39%)	0,021*
Chronic kidney disease	1 (3,3%)	2 (6,5%)	>0,900
Chronic liver disease	1 (3,3%)	2 (6,5%)	>0,900
Nicotine dependence	4 (13%)	2 (6,5%)	0,400
Clinical symptoms:			
Fever	20 (67%)	26 (84%)	0,200
Cough	21 (70)	21 (68)	>0,900
Dyspnea	17 (57%)	23 (74%)	0.200
Tachypnea	6 (20%)	15 (48%)	0,039*
CXR result on admission to hospital [in 5-point scale], mean (SD); range	2,7 (1,2); 2–4	3,5 (1); 3–4	0,009*
CT perform—day of illness, mean (SD); range	12 (6); 8–17	12 (8); 6–17	0,600
Average oxygen saturation (%), mean (SD); range	94 (3); 92–97	82 (8); 78–88	<0,001*
Laboratory test results average, mean (SD); range			
CRP (> 6 mg/L)	40 (43); 7–60	128 (105); 47–178	<0,001*
PCT (> 0,05 ng/mL)	0,21 (0,47); 0,01–0,15	0,73 (1,99); 0,12–0,41	<0,001*
Ferritin (>291 ng/mL)	904 (1272); 292–815	1093 (791); 457–1425	0,100
D-dimers (>500 ng/mL)	2135 (3807); 404–1820	9420 (20144); 1527–3948	0,002*
Lymphocyte count (< 1 x 10^3^/μL)	1,36 (0,57); 0,9–1,8	1,09 (0,65); 0,7–1,4	0,042*
Lymphocyte/Neutrophil index	0,41 (0,21); 0,26–0,47	0,2 (0,17); 0,09–0,29	<0,001*
Neutrophil count (> 7 x 10^3^/μL)	3,72 (1,52); 2,81–4,44	6,48 (2,93); 4,41–8,18	<0,001*
LDH (>246 U/L)	306 (103); 246–361	545 (403); 391–513	<0,001*
ALT (> 48 IU/L)	44 (38); 20–51	59 (42); 26–85	0,110
ICU admission	1 (3,3%)	9 (29%)	0,012*
Respiratory support	2 (6,7%)	14 (45%)	0.002*
High-flow oxygen therapy	1	5	
Respiratory therapy	1	9	
Duration of hospitalization (days), mean (SD); range	20 (10); 15–22	24 (15); 16–30	0,200
Death	1 (3,3%)	11 (35%)	0.001*

**Table 2:** CT results, comparison of severe and non-severe group.

Feature	Total (n = 61)	Severe group (n = 31)	Non-severe group (n = 30)	P
**GGO (%),**	22,8 (15,17); 3,9–78,73	31,42 (15,45); 12,2–78,73	13,89 (8,21); 3,9–38,88	<0,001*
mean (SD); range				
**Consolidation (%),** mean (SD); range	7,65 (8,7); 0,64–58,4	11,85 (10,38); 3,44–58,4	3,32 (2,64); 0,64–12,1	<0,001*
**Total lesion = GGO+consolidation (%),** mean (SD); range	30,45 (20,26); 4,54–99,8	43,27 (19,33); 21,02–99,8	17,21 (10,28); 4,54–48,1	<0,001*
**Emphysema (%),** mean (SD); range	4,19 (5,31); 0–16,59	2,72 (4,34); 0–16,59	5,7 (5,84); 0–13,42	0,016*
**Pulmonary artery/aorta ratio,** mean; range	0,9; 0,8–1,1	0,9; 0,8–1,1	0,9; 0,8–1,1	0,400
**Number of lobes involved (%)**				<0,001*
**1**	1 (1,6)	0 (0)	1 (3,3)	
**2**	1 (1,6)	0 (0)	1 (3,3)	
**3**	8 (13,1)	0 (0)	8 (26,7)	
**4**	3 (4,9)	0 (0)	3 (10)	
**5**	48 (78,7)	31 (100)	17 (56,7)	
**Bilateral (%)**	60 (98,4)	31 (100)	29 (96,7)	0,500
**Crazy pathing (%)**	9 (14,8)	7 (22,6)	2 (6,7)	0,150
**Distribution**				
**Peripheral (%)**	24 (39,3)	5 (16,1)	19 (63,3)	0,002*
**Central (%)**	0 (0)	0 (0)	0 (0)	
**Mixed (%)**	37 (60,7)	26 (83,9)	11 (36,7)	<0,001*
**Subpleural**	15 (24,6)	7 (22,6)	8 (26,7)	>0,900

The analysis revealed a significantly higher severity of changes in type of ground-glass opacities and consolidation in patients with severe disease than in those with non-severe disease. All five lobes were involved in 100% of the patients with severe disease, all of them had consolidation on CT scan. The changes in mixed type (central 2/3 of lung + peripheral 1/3 distal part of lung) were more prevalent than in peripheral types alone.

In the study we researched the correlation between the intensity of particular lesions (GGO, consolidation, total = GGO + consolidation, emphysema) in CT and respective features in severe and non-severe groups. Statistical significance in the non-severe group was found for sex, tachypnea, the number of points in CXR on admission as well as for laboratory parameters: CRP, D-dimer, ALT, lymphocyte count and lymphocyte/neutrophil ratio However, in the severe group, a correlation was only found for sex, creatinine level and death. [Tables 3–5].

**Table 3:** Analysis of the correlation between severity of GGO in CT and respective features in severe and non-severe groups (P-value).

Feature	Severe group	r	Non-severe group	r
**Symptoms duration**	0,904	-0,023	0,914	0,021
**Age**	0,656	0,083	0,704	0,072
**Sex**	0,17	effsize 0,252	0,001*	effsize 0,572
**Tachypnea**	0,188	effsize 0,241	0,009*	effsize 0,464
**CRP**	0,157	0,260	0,011*	0,460
**PCT**	0,271	0,210	0,257	0,250
**D-dimer**	0,470	0,150	0,023*	0,450
**Ferritin**	0,617	0,120	0,414	0,200
**ALT**	0,508	-0,130	0,133	0,290
**LDH**	0,237	0,230	0,176	0,270
**Lymphocyte count**	0,094	-0,320	0,005*	-0,510
**Lymphocyte/neutrophil ratio**	0,076	-0,330	0,024*	-0,420
**Neutrophil count**	0,257	0,220	0,973	-0,007
**Creatinine**	0,026*	0,400	0,272	0,210
**CXR 5**	0,706	0,072	0,054	0,400
**Death**	0,003*	effsize 0,526	0,333	effsize 0,221

**Table 4:** Analysis of the correlation between severity of consolidation in CT and respective features in severe and non-severe groups (P-value).

Feature	Severe group	r	Non-severe group	r
**Symptoms duration**	0,193	0,024	0,220	0,023
**Age**	0,221	-0,023	0,397	0,160
**Sex**	0,055	effsize 0,349	0,011*	effsize 0,466
**Tachypnea**	0,767	effsize 0,057	0,004*	effsize 0,530
**CRP**	0,580	0,100	0,092	0,310
**PCT**	0,981	-0,005	0,369	0,200
**D-dimer**	0,852	0,039	0,028*	0,440
**Ferritin**	0,840	-0,047	0,754	0,077
**ALT**	0,618	-0,095	0,027*	0,410
**LDH**	0,989	0,003	0,265	0,220
**Lymphocyte count**	0,421	-0,160	0,055	-0,360
**Lymphocyte/neutrophil ratio**	0,624	-0,095	0,104	-0,310
**Neutrophil count**	0,906	-0,023	0,983	-0,004
**Creatinine**	0,760	-0,057	0,386	0,160
**CXR 5**	0,699	0,074	<0,001*	0,630
**Death**	0,509	effsize 0,122	0,563	effsize 0,116

**Table 5:** Analysis of the correlation between severity of GGO+consolidation in CT and respective features in severe and non-severe groups (P-value).

Feature	Severe group	r	Non-severe group	r
**Symptoms duration**	0,955	0,011	0,455	0,140
**Age**	0,480	-0,130	0,593	0,100
**Sex**	0,034*	effsize 0,381	0,003*	effsize 0,542
**Tachypnea**	0,093	effsize 0,305	0,005*	effsize 0,516
**CRP**	0,081	0,320	0,032*	0,390
**PCT**	0,174	0,260	0,356	0,200
**D-dimer**	0,383	0,180	0,043*	0,410
**Ferritin**	0,470	0,170	0,716	0,089
**ALT**	0,481	-0,130	0,051	0,370
**LDH**	0,182	0,260	0,198	0,260
**Lymphocyte count**	0,070	-0,340	0,015*	-0,450
**Lymphocyte/neutrophil ratio**	0,125	-0,290	0,047*	-0,370
**Neutrophil count**	0,592	0,100	0,994	-0,002
**Creatinine**	0,307	0,190	0,336	0,180
**CXR 5**	0,279	0,200	0,005*	0,560
**Death**	0,016*	effsize 0,430	0,248	effsize 0,222

Correlation between particular changes in CT and the presence of comorbidities or any other than dyspnea, clinical symptoms (dyspnea, fever, cough) has been checked and no statistical significance was found. Severity of emphysema with the above listed features has been also analysed. Statistical significance has only been found between tachypnea and creatinine concentration in the severe group.

**Fig 2:**
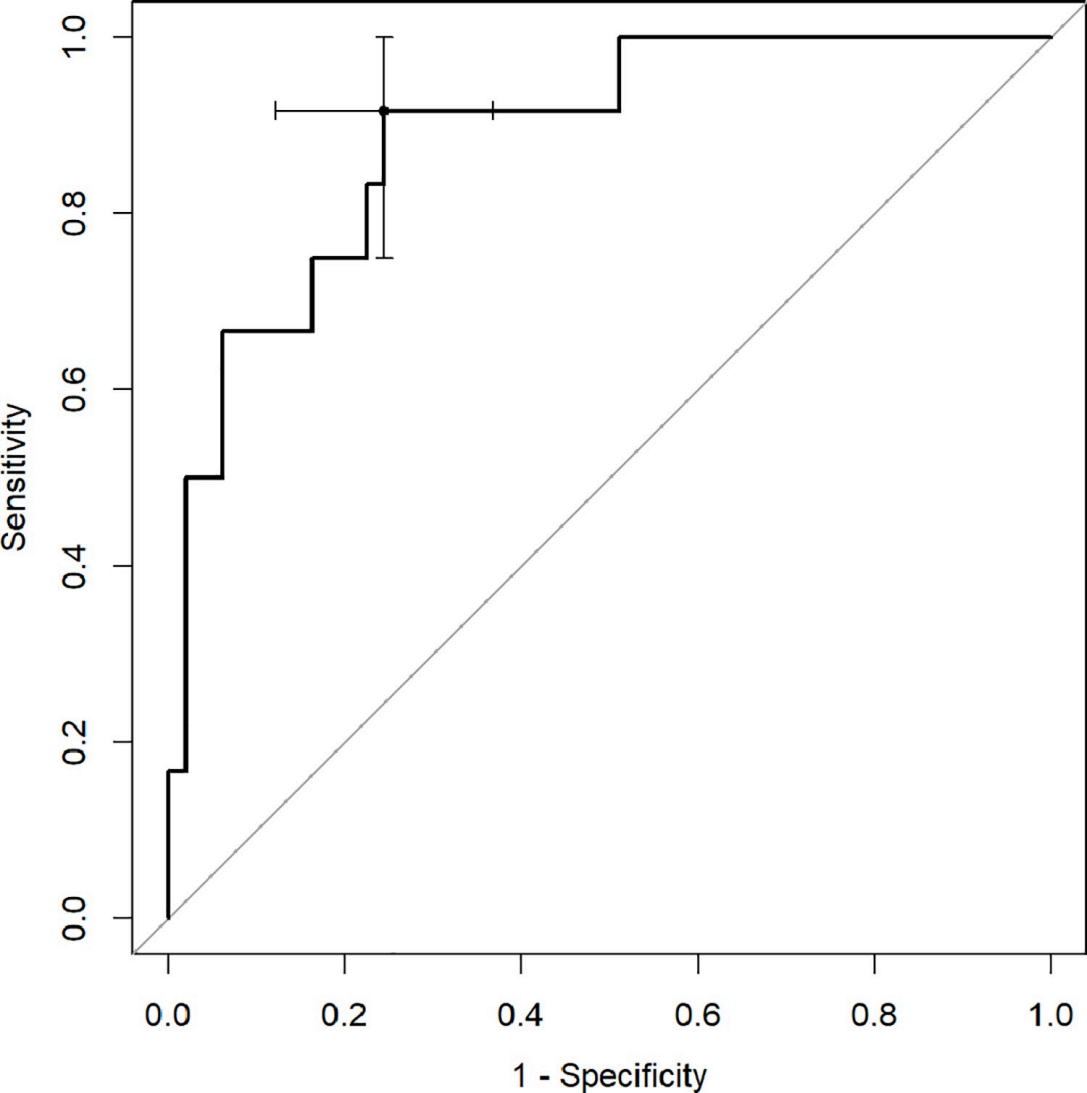
Receiver operating characteristic (ROC) curves analysis of GGO score for assessing the risk of death in COVID-19.

**Fig 3:**
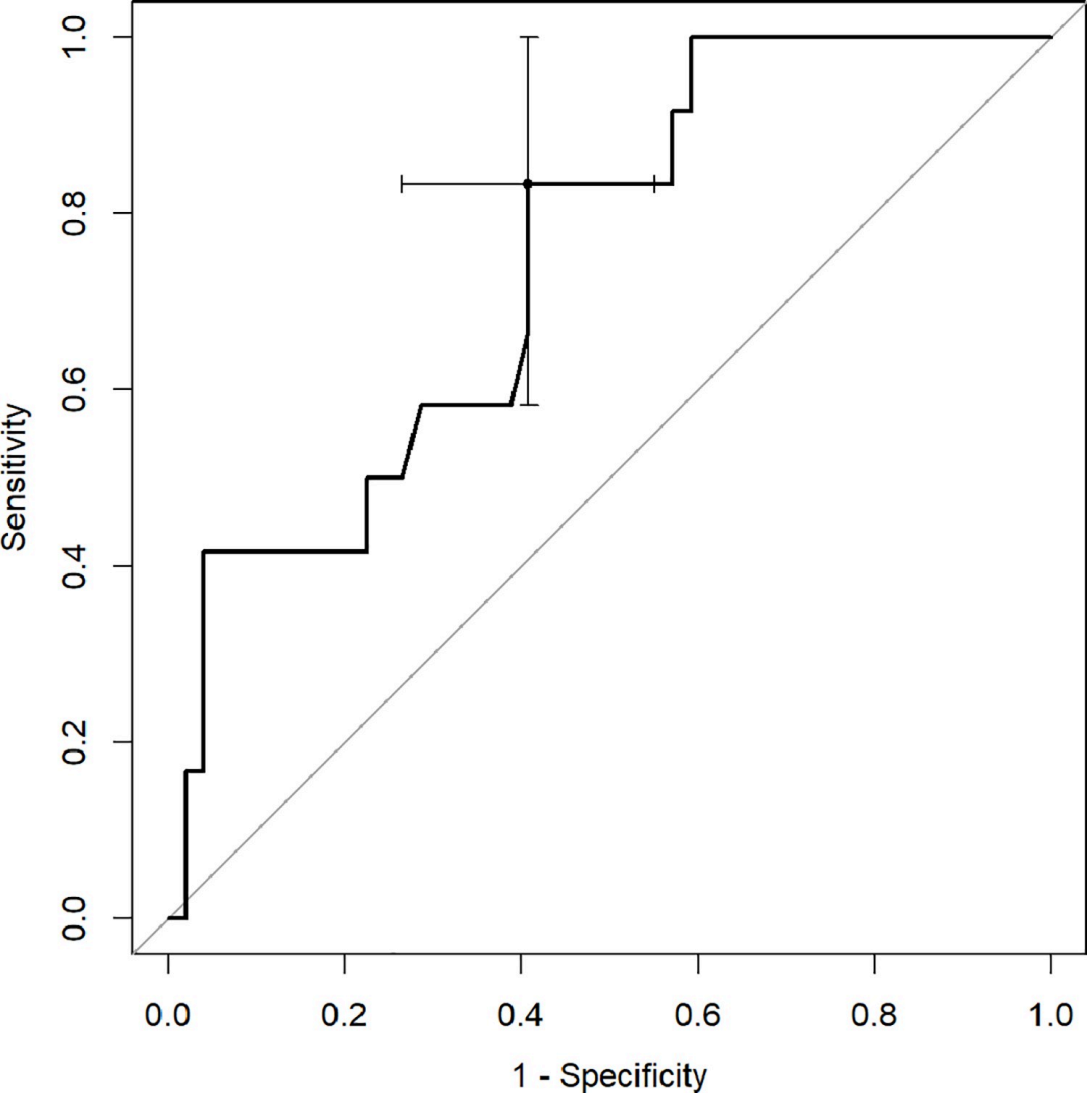
Receiver operating characteristic (ROC) curves analysis of consolidation score for assessing the risk of death in COVID-19.

**Fig 4:**
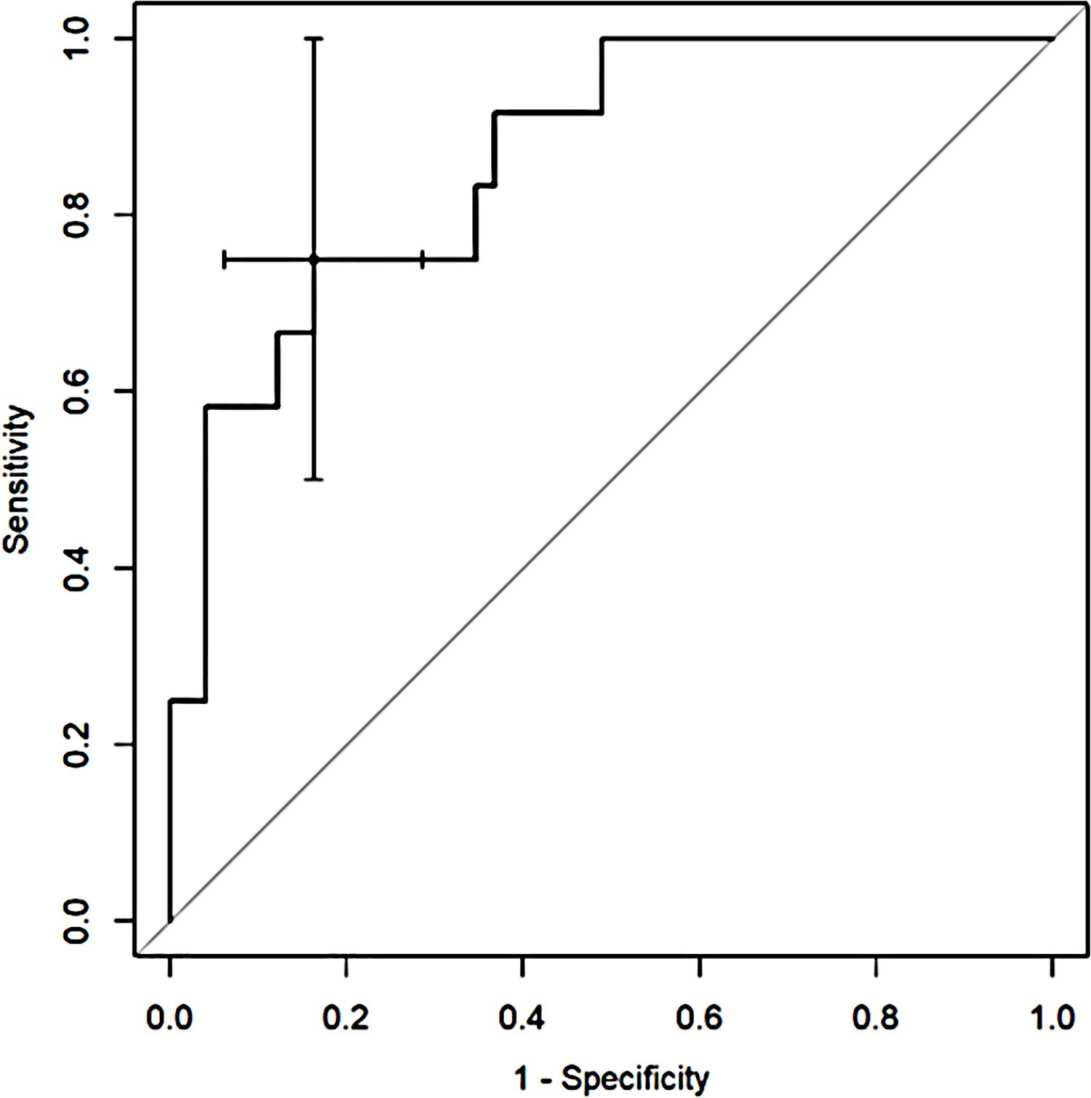
Receiver operating characteristic (ROC) curves analysis of total score for assessing the risk of death in COVID-19.

**Fig 5:**
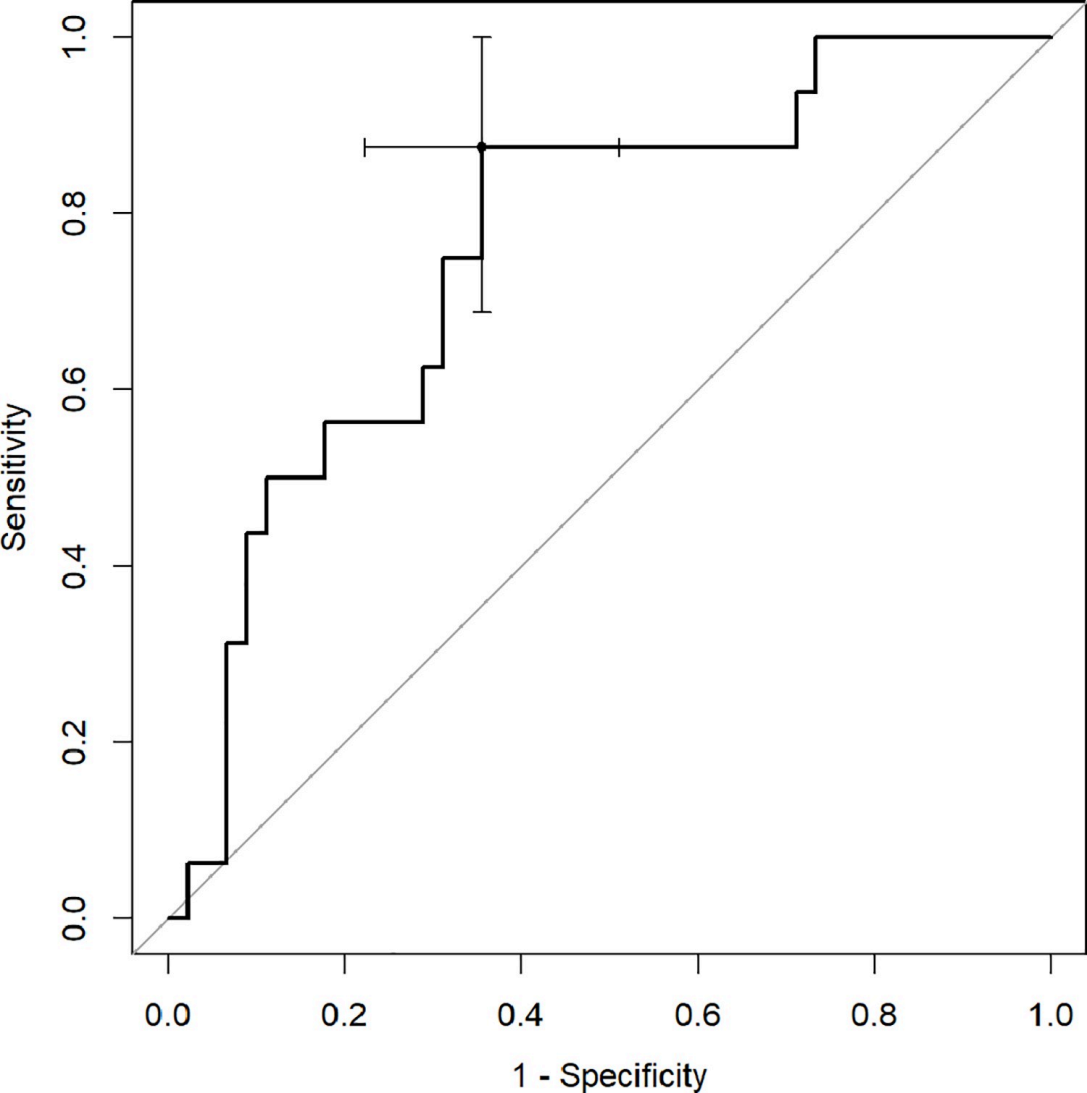
Receiver operating characteristic (ROC) curves analysis of GGO score for assessing the risk of respiratory support in COVID-19.

**Fig 6:**
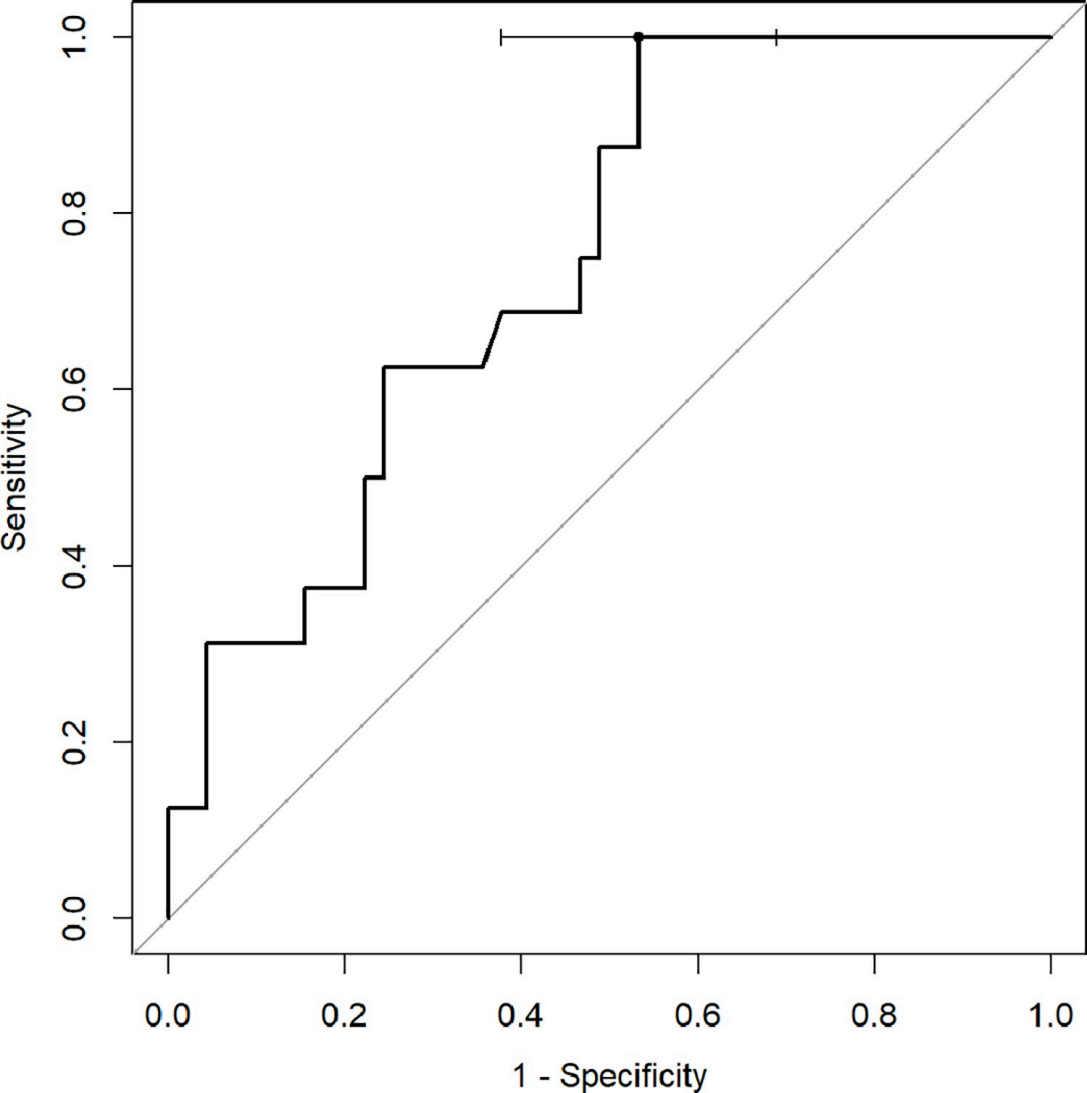
Receiver operating characteristic (ROC) curves analysis of consolidation score for assessing the risk of respiratory support in COVID-19.

**Fig 7:**
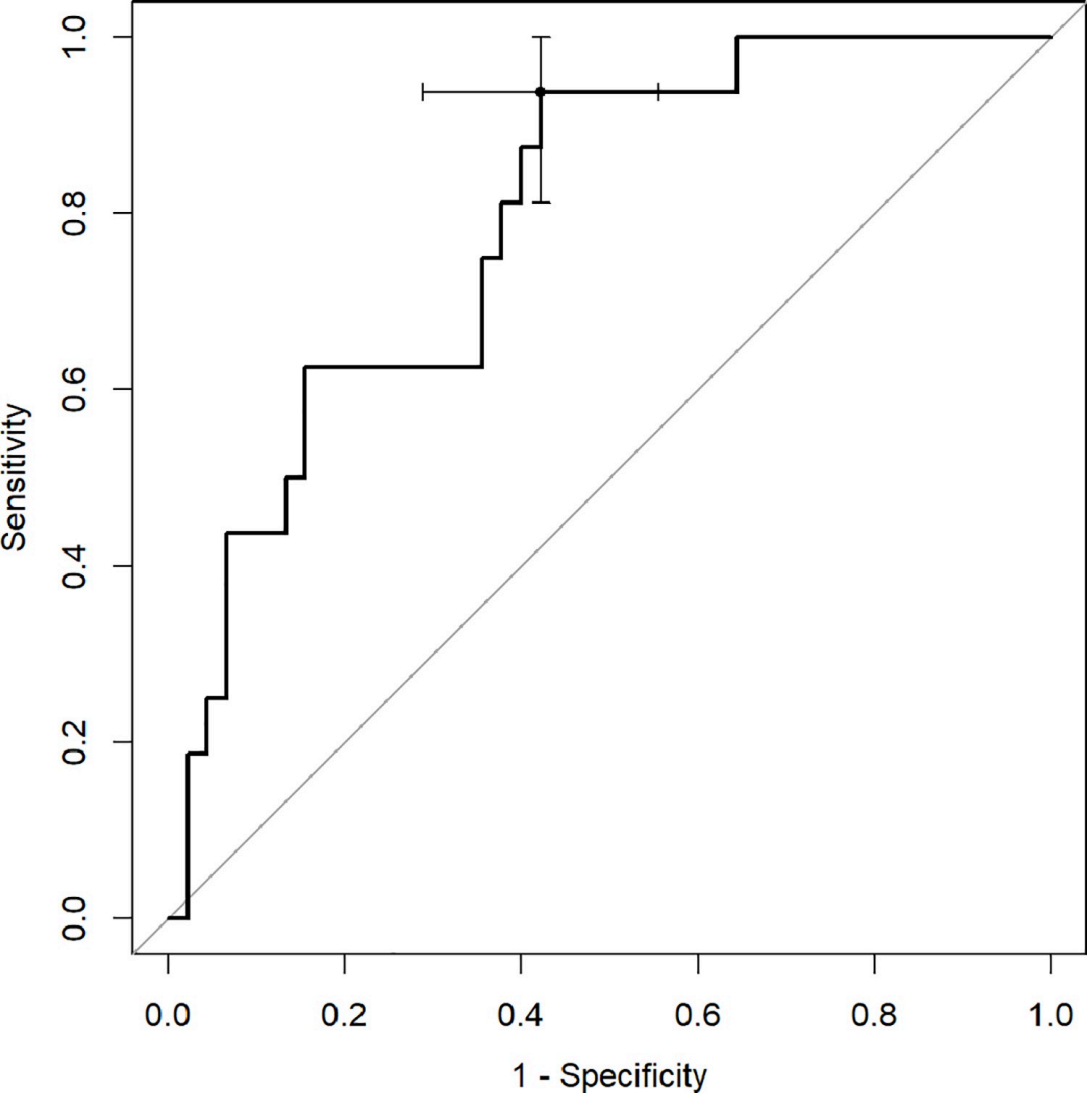
Receiver operating characteristic (ROC) curves analysis of total score for assessing the risk of respiratory support in COVID-19.

ROC analysis with Youden index was used to determine diagnostic capabilities of CT quantitative parameters to predict death and the need for respiratory support. The percentage of inflamed lung parenchyma, which correlates with a higher risk of death (Figs 2–4), as well as respiratory support (high-flow oxygen therapy, mechanical ventilation) (Figs 5–7), was assessed. Optimal cut-off points are as follow:

**Risk of death.**

- GGO 24.3% (AUC 0.8878, 95% CI 0.7889–0.9866; sensitivity 91.7%, specificity 75.5%),
- consolidation 5.6% (AUC 0.7466, 95% CI 0.6009–0.8923; sensitivity 83.3%, specificity 59.2%),
- total 37.8% (AUC 0.8622, 95% CI 0.7525–0.972; sensitivity 75%, specificity 83.7%);

**Risk of respiratory support.**

- GGO 18.7% (AUC 0.7611, 95% CI 0.6268–0.8954; sensitivity 87.5%, specificity 64.4%),
- consolidation 3.88% (AUC 0.7438, 95% CI 0.6146–0.8729; sensitivity 100%, specificity 46.7%),
- total 23.5% (AUC 0.7931, 95% CI 0.673–0.9131; sensitivity 93.8%, specificity 57.8%).

**Fig 8:**
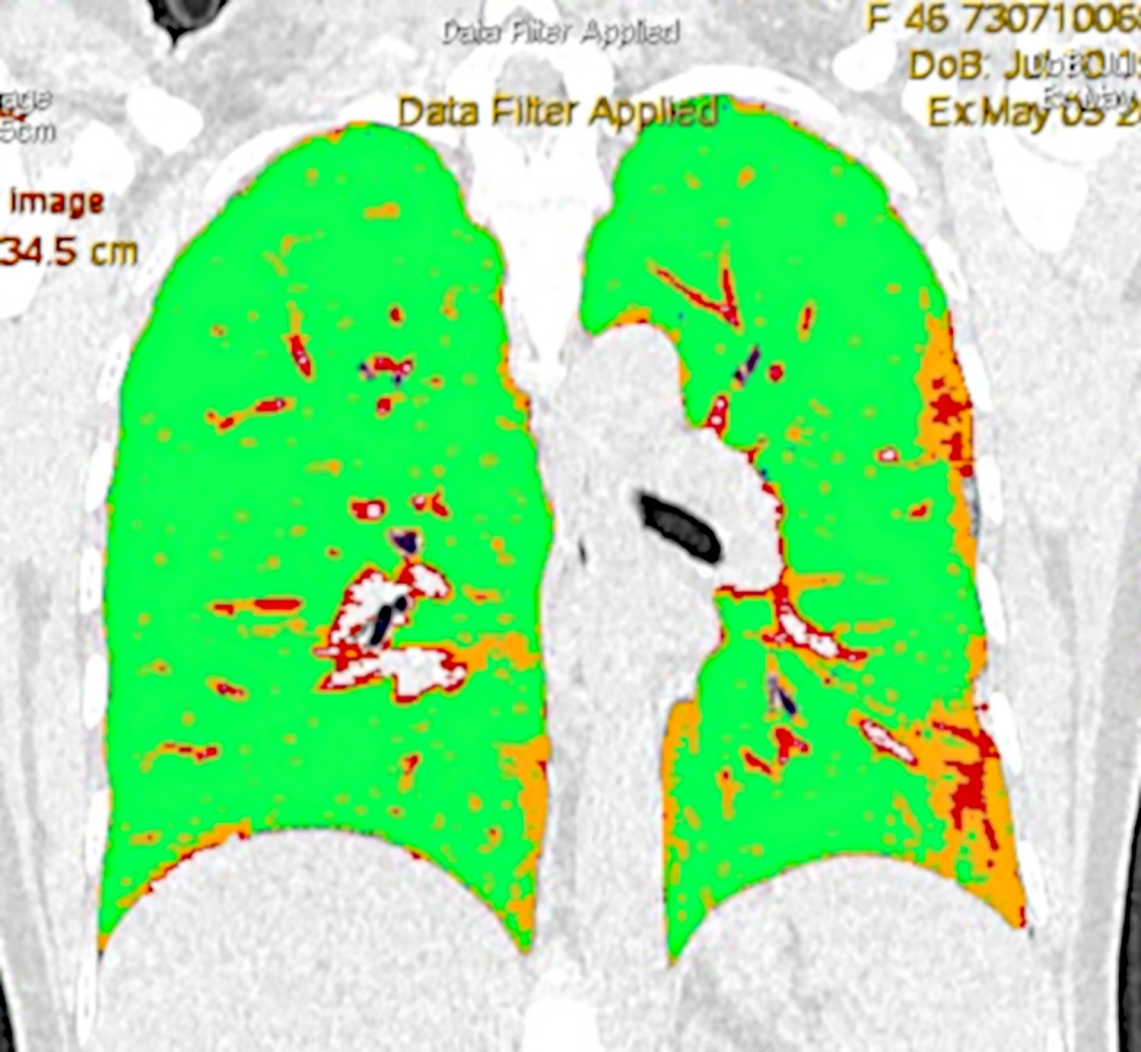
Quantitative computed tomography (QCT) with Thoracic VCAR software in the non-severely ill patient: Orange color—ground-glass opacities 12,2597% total lung volume involved; red color—consolidation 6,2129% total lung volume involved); blue color–emphysema 0,0940% total lung volume involved; green color—normal parenchyma 81,4334% total lung volume.

**Fig 9:**
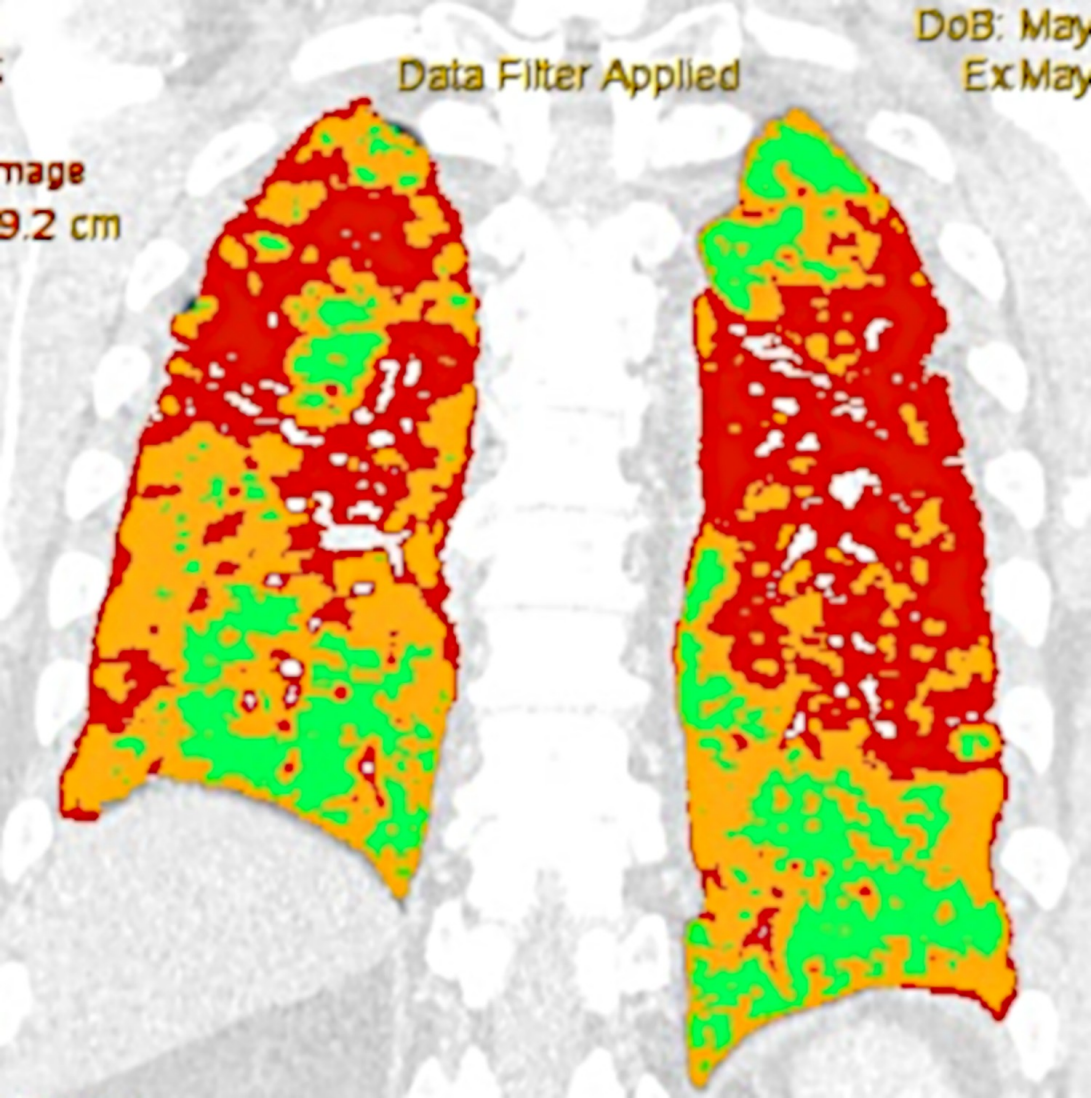
Quantitative computed tomography (QCT) with Thoracic VCAR software in the severely ill patient: Orange color—ground-glass opacities 45,3011% total lung volume involved; red color—consolidation 28,0170% total lung volume involved); blue color–emphysema 0,6004% total lung volume involved; green color—normal parenchyma 26,0815% total lung volume.

Quantitative computed tomography (QCT) with Thoracic VCAR software in the non-severely and severely ill patient are shown in the Figs 8 and 9.

## Discussion

Chest computed tomography is very useful in the screening, diagnosis and follow-up of COVID-19 patients. Most of the research conducted so far has focused on the qualitative assessment of images, analysing location and a type of changes in order to increase the diagnostic sensitivity of the method [16, 17]. CT images of COVID-19 are often manually evaluated by radiologists, which is subjective and essentially focuses on confirming a diagnosis or assessing the morphology of changes. In their study Grassi R. et al. noted that software programs to provide automatic CT quantitative measurement in COVID-19 patients can be used in clinical practice to assist radiologists diagnoses but when using them there might appear mistakes demanding manual evaluation [18].

To date, not many researchers have addressed the quantitative assessment of pathological changes in COVID-19 pneumonia which involves a separate type of imaging. Our research focuses on the quantitative assessment of changes in lung parenchyma correlated with clinical data. Such insight reveals new diagnostic value which offers considerable support in clinical decisions. The efficiency of computer programs to precisely calculate the volume of affected areas has been proven in many studies [18, 19]. This is useful for evaluating disease progression/regression and is valuable in predicting poor outcomes of COVID-19. The method is fast, standardised and the result is consistently repeatable [20]. Lyu P. et al. showed that combined quantitative indicators in CT could achieve high sensitivity(92%) specificity (87%) and accuracy (90%) in distinguishing critical cases from severe cases [21].

Li et al. were among the first scientists to conduct a visual, quantitative analysis of lung damage in a group of 78 patients (including 8 severe critical patients) with COVID-19 who underwent a CT scan on admission to the hospital. The images were assessed by radiologists. The final result was reached by adding up the five lobe scores (acute lung inflammatory lesions were assessed using a scale of 0–4). The researchers confirmed that in this group all 5 lobes were involved in all cases and the patients obtained a significantly higher median total severity scores than common type (p<0.001) [22]. In their study, Sun et al. noted that severe patients (23/84) had a significantly higher GGO score, consolidation score, total lesion score and consolidation percentage but lower GGO percentage (relative to total lesion volume) compared to non-severe patients. In contrast to the earlier research, 73.9% of patients in this group had 5 lobes involved. [23]. In our research, all the patients from the severe group had lesions (GGO +/- consolidation) in all the lobes, which was statistically significant compared to the non-severe group. In addition to GGO and consolidation, emphysema volume and pulmonary artery/aorta ratio were studied. GGO and consolidation were statistically present more often in the severe group compared to the non-severe group, adversely than in the case of emphysema. There were no differences in pulmonary artery/aorta ratio as this parameter was within the normal range in most patients. Additionally, it is worth mentioning that the patients from the first group, although younger, had a higher number of comorbidities and had higher inflammatory markers, D-dimers and LDH activity, but lower lymphocyte counts and lymphocyte/neutrophil index. The prediction value of these markers is not the subject of this research and has been discussed by many other researchers [24, 25].

The first cohort study to predict outcomes in patients with COVID-19 using non-invasive quantitative CT measurements was conducted by Liu et al. In the research in the group of 134 patients (including 19 with severe disease), they detected that the change to the CT image from day 0 to day 4 predicts progression to severe illness [26]. Yu Q. et al. found that advanced age and greater consolidation in the upper lungs on admission were associated with higher rates of admission to the intensive care unit (ICU) and acute respiratory failure [27]. Colombi D. et al. proved the value of quantification of well-aerated lung (WAL) in predicting the need to apply intensive therapy or predicting death [20, 28]. Colombi D. et al. have shown that a global percentage of WAL parenchyma below 71% in case of software-based evaluation or 73% for visual quantification and WAL volume less than 2.9 L were predictors of ICU admission and death. The median value of WAL using visual scoring in patients admitted to ICU was 53% and in software-based scoring 57%, while in those without ICU admission it was 80% and 78% respectively [28]. Significantly different results were published by Nishiyama A. et al; this research was conducted in patients with ARDS diagnosed in the course of COVID-19, hospitalised in intensive care units. WAL showed a positive correlation with 28-day survival (P = 0.020), and lung volumes below -900 HU correlated positively with 28-day survival and ICU survival, respectively (P = 0.028, 0.017). Survival outcome was better in case of WAL ≥40% compared to WAL <40% (P = 0.039) [29].

Our study resulted in higher scores in comparison to the research by Colombi D. et al., but significantly lower scores than those obtained by Nishiyama A. et al., which we associate with different populations and other assumptions for severity criterion and the time the CT scans were performed. We observed that the involvement of at least 37.8% or more, 24.3% by GGO, was a significant mortality predictor. The differences in researches might also result from applying other definitions of normal lung density: the interval between −950 and −700 HU in the Colombi study, but −900 and −501 HU in the Nishiyama study and our study range for non-parenchymal involvement (WAL) was -950 to -700 HU. The threshold attenuation range between -500 and -700 HU in the Shin study and range between -700 to 0 HU in the Matsuoka study has been adopted for the evaluation of the extent of interstitial lung disease, and studies found that it had an excellent correlation with the decrease in diffusing capacity of the lungs for carbon monoxide and pulmonary function tests [30, 31]. In the Nishiyama study, we should also note the high percentage of patients who left ICU alive which is not observed in Poland (in the group studied, less than 1/10 patients who were mechanically ventilated survived). The higher rate of survival might be connected with an easier course of the disease or with the early application of mechanical ventilation.

The prognostic value of QCT was also researched by Lanza E. et al. The authors revealed that compromised lung volume is the best predictor for oxygenation support, intubation and in-hospital death. In case of intubation, the other independent factor was the level of urea on admission. The risk of death was also associated with age, C-reactive protein concentration, cancer and chronic kidney disease. It was identified that %CL (compromised lung) values in the range of 6–23% correlated with increased risk of oxygen support and a value over 23% with an increased risk of intubation. %CL had a negative correlation with the PaO_2_/FiO_2_ ratio. One of the conclusions of the study is that QCT might be useful for predicting which patients will need mechanical ventilation soon [20]. The results of our research are similar to those mentioned above. We determined that 23.5% of lung involvement, including 18.7% by GGO, was connected with significantly higher risk of respiratory support, that is to say high-flow oxygen therapy as a minimum. What is interesting, the significant predictive importance factor was only 3.88% involvement of the lung by consolidation.

Several biomarkers have been evaluated to assess the severity of COVID-19 pneumonia and to predict prognosis in patients with ARDS, however they are not accurate enough to determine the further course of the disease [2, 3]. In this study, the correlation between the severity of particular lesions in CT and the level of particular biomarkers was mainly found in the non-severe group. However, clear correlation between lung involvement and death was found in the severe group. This means that in severely ill patients, after reaching a particular critical value, further progression of the disease will not influence the growth of inflammatory parameters, D-dimers, LDH or the decrease in the lymphocyte count. Park B. et al. revealed that CRP in the mild pneumonia group and normally aerated lung proportion volume in the severe pneumonia group were independently associated with critical event-free survival in patients with COVID-19 [32].

The measurement of lung involvement is the first independent factor enabling prediction not only of the risk of death but also of the need to use oxygen support or mechanical ventilation. The papers published to date concerning severely ill patients involved relatively small groups, with different selection criteria and the research results defining correlation of well-aerated lung volume and prognosis not being fully coherent, thus there is a need for further research. CT lung attenuation could be affected by congestive heart failure and pulmonary (micro)embolism, which determine significant alterations of the lung density histogram, such as a shift to higher HU values or a reduction in skewness and kurtosis.

Nevertheless, relatively easy-to-use computer programs enabling the quantitative analysis of CT scans in patients with COVID-19 can provide clinicians with valuable information on death prediction as well as on decisions to intensify treatment.

## Conclusions

It is important to identify patients with respiratory failure risk factors in the course of COVID-19 at an early stage and focus on prevention and treatment efforts. Quantitative computed tomography is an excellent tool for assessing lung parenchyma involvement (GGO and consolidation). When correlated with clinical data, it simplifies decisions to introduce the right oxygen support therapy or to admit a patient to the intensive care unit. QCT can make an independent and simple screening tool to assess the risk of death, regardless of clinical symptoms. Usefulness of QCT to predict the risk of death is higher than to assess the indications for respiratory support.
